# Relationship between breast cancer and metformin: A bibliometric review

**DOI:** 10.1097/MD.0000000000047868

**Published:** 2026-02-28

**Authors:** Zhaoning Wang, Fangrui Zhao, Linlin Wang

**Affiliations:** a Shandong First Medical University and Shandong Academy of Medical Sciences, Shandong Cancer Hospital and Institute, Jinan, Shandong Province, China.

**Keywords:** bibliometric analysis, breast cancer, diabetes mellitus, metformin

## Abstract

**Background:**

A complex relationship exists between diabetes, particularly type 2 diabetes, and breast cancer. Metformin can help reduce the incidence of cancer and the risk of death in individuals with type 2 diabetes. This study utilized bibliometrics to analyze the potential impact of diabetes drugs on the treatment of breast cancer, aiming to reveal research trends and provide references for future research directions.

**Methods:**

This bibliometric study analyzed the intersection of diabetes, breast cancer, and metformin using data from the Web of Science Core Collection and PubMed (2004–2024). A total of 1080 relevant publications were included.

**Results:**

An initial slow growth in publications was noted, followed by a surge starting in 2008. The USA and China led these research efforts, with Goodwin PJ having the highest number of publications. The journal Diabetes Care was the most co-cited, with keywords such as “breast cancer,” “risk,” “metformin,” and “growth” frequently appearing. The analysis identified 4036 institutions and 5214 active authors in the field.

**Conclusions:**

Despite growing attention to the role of diabetes medications such as metformin in breast cancer treatment, research remains limited to mechanistic studies and retrospective clinical analyses. The optimal dose for clinical use remains unclear, which highlights the need for further experimental studies. This study provides valuable insights for both academic and clinical research, supporting the potential use of metformin to manage the relationship between diabetes and breast cancer.

## 1. Introduction

Breast cancer is the most common malignancy in women, accounting for 30% of all new cancer cases, and it is increasing at a rate of approximately 0.5% per year.^[1]^ Diabetes is a metabolic disorder characterized by elevated blood sugar levels. Type 2 diabetes, which accounts for >90% of the total number of patients with diabetes, is characterized by insulin resistance, the reduced sensitivity of fat and muscle tissues to insulin. This results in insufficient insulin secretion, which leads to increased compensatory insulin production.^[2]^

The association between diabetes and certain cancers was first documented a century ago. Diabetes is widely recognized as a risk factor for many types of cancer, including endometrial and pancreatic cancers. Preclinical and clinical studies have revealed a complex relationship between diabetes, particularly type 2 diabetes, and breast cancer. Approximately 16% of patients with breast cancer have diabetes. In addition, the 2 main risk factors for type 2 diabetes, increasing age and being overweight, are also strongly linked to the development of breast cancer.^[3]^ Diabetes is considered an independent risk factor for breast cancer.^[4–7]^

Recently, metformin has received increasing attention in the field of breast cancer treatment because of its potential antitumor effects. Metformin, a commonly prescribed medication for individuals with type 2 diabetes, inhibits the AMP-activated protein kinase (AMPK) enzyme and is considered a protective factor that reduces the incidence of cancer and the risk of death in people with type 2 diabetes.^[8]^ This action prompts muscle tissues to absorb glucose from the bloodstream. Recent studies have identified *liver kinase B1 (LKB1*), a protein kinase, as an upstream regulator of AMPK. *LKB1* is known for its role as a tumor suppressor. The activation of AMPK by both metformin and physical activity is *LKB1*-dependent; this provides insight into the preventative effects of exercise against the development and recurrence of certain types of cancer.^[9]^ In addition, metformin may have a positive impact on progression-free survival in ovarian cancer patients,^[10]^ prognosis in breast cancer patients, and overall survival in patients with metastatic non-small cell lung cancer^[11]^ and nasopharyngeal carcinoma.^[12]^

This study applied bibliometrics to conduct statistical and quantitative analyses of the potential impact of diabetes drugs on the treatment of breast cancer, with the aim of revealing research trends and providing references for future research directions.

## 2. Methods

### 2.1. Ethics approval and consent to participate

This study does not involve any experiments on humans or animals. The data used were obtained from previously published literature/databases and did not include any identifiable human information. Accordingly, ethics committee approval and informed consent were not required.

### 2.2. Data sources

The relevant literature was systematically collected from the Web of Science Core Collection (WOSCC) and PubMed following established criteria for inclusion, and the data were saved in a full-record plain text format. Any discrepancies were resolved through collaborative discussions. Subsequently, duplicate records were removed using the built-in deduplication features of Cite Space 6.3. R1, and visualizing scientific landscapes (VOS) Viewer 1.6.20. Statistical analysis was performed to evaluate the yearly publication output and the spectrum of publication types.

### 2.3. Search strategy

Data were sourced from the WOSCC and PubMed databases. To account for variations in the expressions of keywords, WOSSCC uses a (Topic) search term connected with “AND,” while PubMed uses free words connected with “OR” before being connected by “AND.” The search formulas are presented in Table S1, Supplemental Digital Content, https://links.lww.com/MD/R472. Our study had no restrictions on the start date of the search; the end date was set to December 2024. The language of the included studies was restricted to English.

### 2.4. Parameter setting

The documents were imported into CiteSpace for co-occurrence and clustering analysis. The time slice of keyword nodes was 1 year, and the threshold value was top N = 25, indicating that the top 25 most frequently occurring keywords from each year were filtered out to construct the co-occurrence network for that year. Subsequently, individual networks were synthesized. The modularity score *Q* (ranging from 0 to 1) was employed to assess the network structure, with a value exceeding 0.3 indicating a well-structured network. The silhouette score *S* (ranging from −1 to 1) was used to evaluate the trustworthiness of clustering mapping. Values above 0.5 indicate reasonable clustering, whereas those exceeding 0.7 signify a highly trustworthy network.^[13]^ The double graph overlay analysis in CiteSpace was conducted using default parameters. In the VOS viewer, adjustments were primarily made to the number of nodes in the graph, whereas the remaining parameters remained at their default values.

### 2.5. Data analysis

A total of 1081 studies were retrieved, with 1080 remaining after deduplication. Using Cite Space 6.3.R1, we performed co-occurrence and clustering analyses on the documents, examining various aspects, such as countries, institutions, authors, journals, co-cited references, and keywords. A network diagram was created, with the links between the nodes symbolizing either appearance or co-citation ties, and the density of these links suggesting a stronger collaboration or a higher level of co-occurrence and co-citation. The magnitude of each node corresponds to its frequency of appearance, or the number of citations received, while the circumference of the circles, marked by thickness and color, reflects the frequency of the term and the timeframe of its citations. VOS Viewer 1.6.20 was used to perform keyword cluster analysis (Figure S1, Supplemental Digital Content, https://links.lww.com/MD/R472). In this visual representation, keywords are presented as nodes with lines connecting them to indicate co-citation relationships. The nodes are color-coded, shaped, and sized to denote the significance of each keyword. In a co-citation network, nodes represent different keywords, and their size indicates the frequency of the keyword. The connecting lines represent co-citation links, while the varying colors of the points and lines distinguish between different clusters or periods. The data retrieval procedure included uploading the data into the Viewer, choosing the option to create a map based on txt file words, and importing the search results from WOSCC and PubMed. Subsequently, irrelevant terms were manually filtered to achieve co-occurrence outcomes.

## 3. Results

### 3.1. Trend analysis of publications

**Figure 1.**
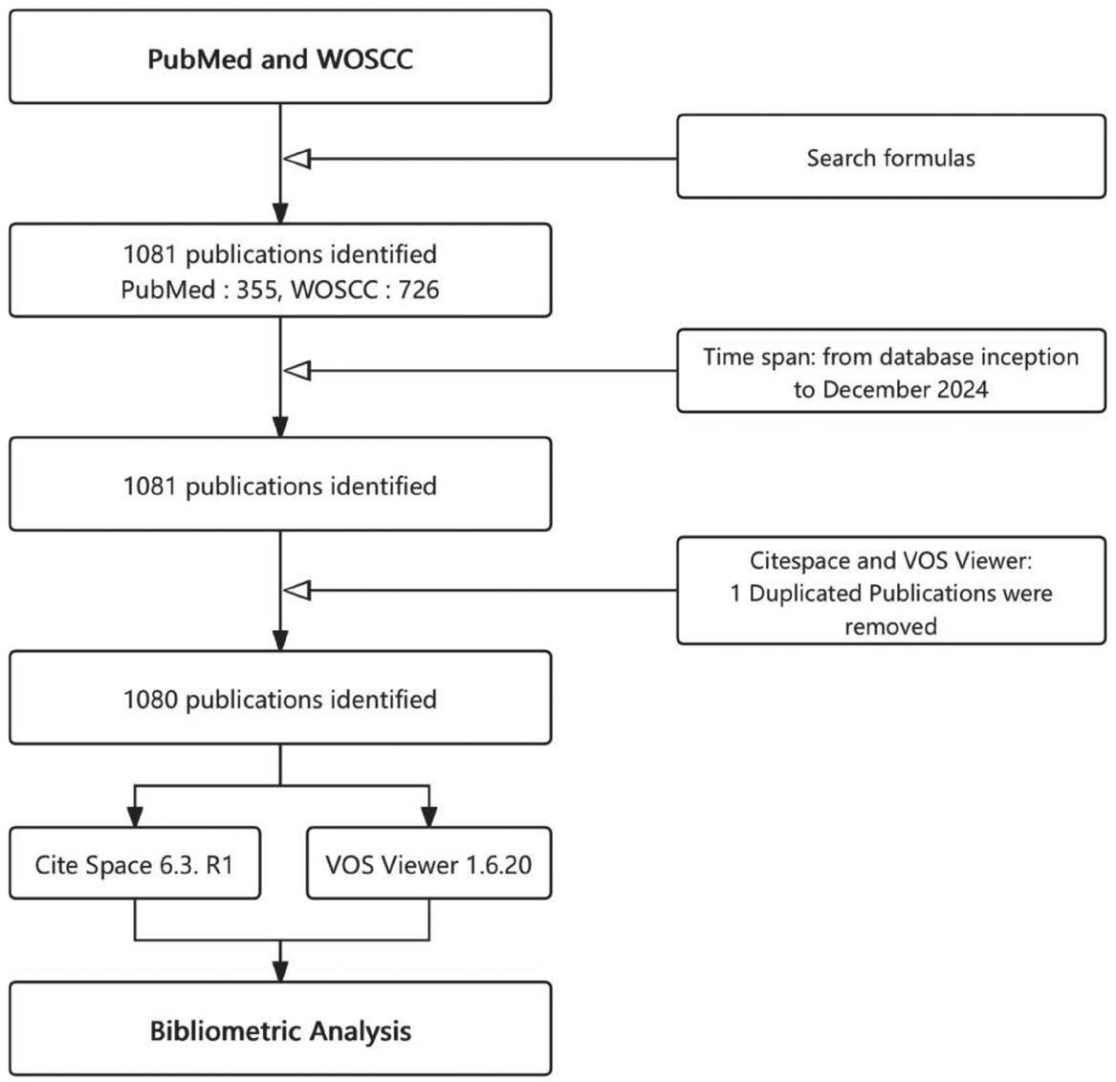
Flowchart of literature selection. VOS = visualizing scientific landscapes, WOSCC = Web of Science Core Collection.

A total of 1080 papers were analyzed from 549 different journals covering January 2004 to December 2024. The screening process is shown in Figure 1. A total of 5214 authors contributed to this study. Overall, the number of these studies has shown a trend of rapid growth and then decline over time. In 2004 and 2008, the number of articles was small, totaling 16. After 2008, the number began to surge, peaking in 2015 with 92 articles.

**Figure 2.**
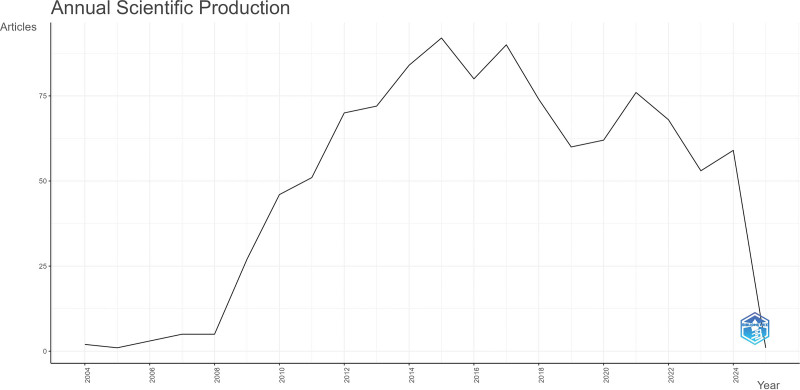
Number of studies from 2004 to 2024.

The number of articles fell after 2015, but grew to a small peak in 2017. Since then, the number of published articles has decreased. The fastest growth occurred between 2008 and 2009. Overall, this trend indicates a growing interest in the prevention and treatment of breast cancer through the mechanisms of action of antidiabetic drugs and their effects, and has attracted more attention from scholars. However, the number of published studies in this field has gradually decreased in recent years. In response, we considered whether studies on the efficacy and safety of diabetes drugs such as metformin have been stymied, leading scholars to turn to other areas (Fig. 2).

### 3.2. Analysis of countries/regions

**Figure 3.**
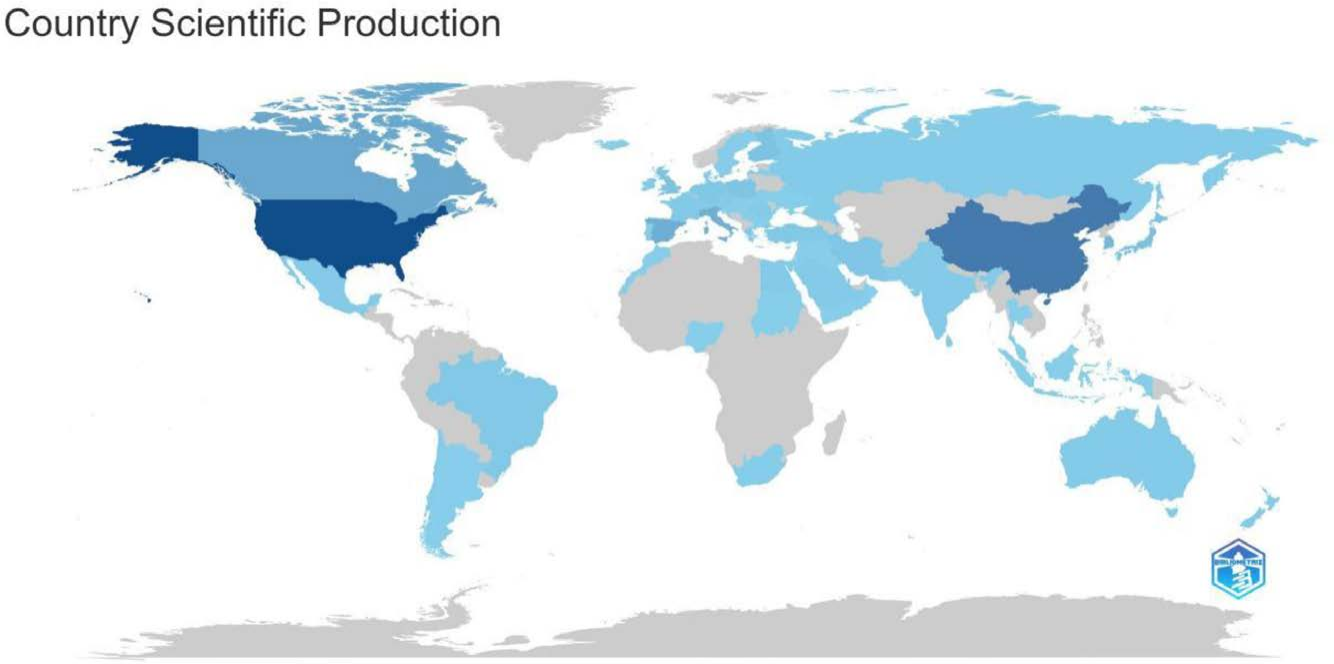
Distribution of relevant research in global regions.

**Figure 4.**
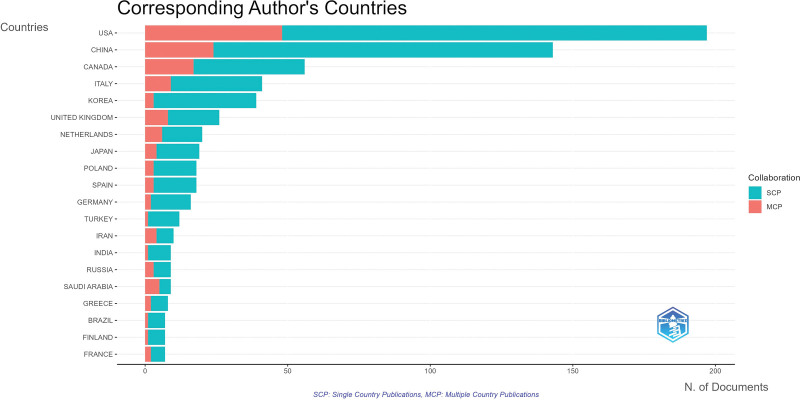
Distribution of the proportion of SCP and MCP research among the top 20 countries in terms of the quantity of studies. MCP = multiple-country publications, SCP = single-country publications.

SCP refer to single-country publications, while MCP refer to multiple-country publications. Then, contributions to the field of diabetes and breast cancer came from 49 countries, of which the top 5 countries were the USA (197 papers: 149 SCP and 48 MCP), China (143 papers: 119 SCP and 24 MCP), Canada (56 papers: 39 SCP and 17 MCP), Italy (41 papers: 32 SCP and 9 MCP), and Korea (39 papers: 36 SCP and 3 MCP). The prominence of the United States and China in the field should be duly acknowledged, given their numerical advantage over other countries (Figs. 3–4).

### 3.3. Analysis of institutions

Between January 2004 and December 2024, 4036 institutions participated in this study and were extensively analyzed through visualization. Table 1 details the top 10 institutions by publication count, with the University of Texas System leading with 28 publications, Harvard University ranking second with 25 publications, and the University of Toronto ranking third with 24 publications.

**Table 1:** Top 10 institutions with the highest number of publications.

	Institutions	Yr	Articles
1	University of Texas System	2008	28
2	Harvard University	2009	25
3	University of Toronto	2006	24
4	Department of Medicine	2008	21
5	McGill University	2006	18
6	University of California System	2012	18
7	UTMD Anderson Cancer Center	2008	18
8	Department of Oncology	2006	16
9	Mayo Clinic	2014	14
10	Sinai Health System, Toronto	2006	14

### 3.4. Analysis of authors and co-cited authors

**Figure 5.**
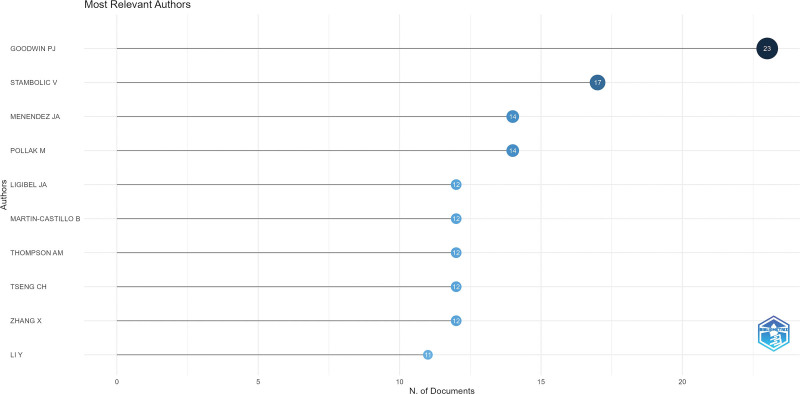
Top 10 scholars in terms of the number of published studies.

**Figure 6.**
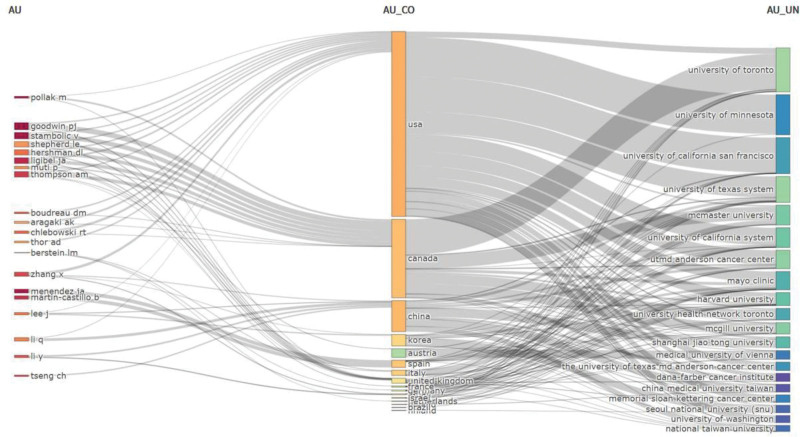
Author, country, and institution.

Over the past 20 years, 5214 authors have contributed to the development of diabetes and breast cancer. Figure 5 displays detailed information on the top 10 authors who have published the most articles in this field, namely Goodwin PJ, Stambolic V, Menendez JA, Pollak M, and Ligibel JA. Figure 6 shows the collaboration between authors, countries, and institutions. The United States cooperates more with the University of Minnesota and the University of California, San Francisco. Canada cooperates more with the University of Toronto and Master University. They primarily work closely with domestic institutions and lack cross-country cooperation. This reflects the small number of global scholars collaborating in this field, as well as the need to further strengthen international research collaborations (Figs. 5–6).

### 3.5. Analysis of journals, co-cited journals, and co-cited references

**Figure 7.**
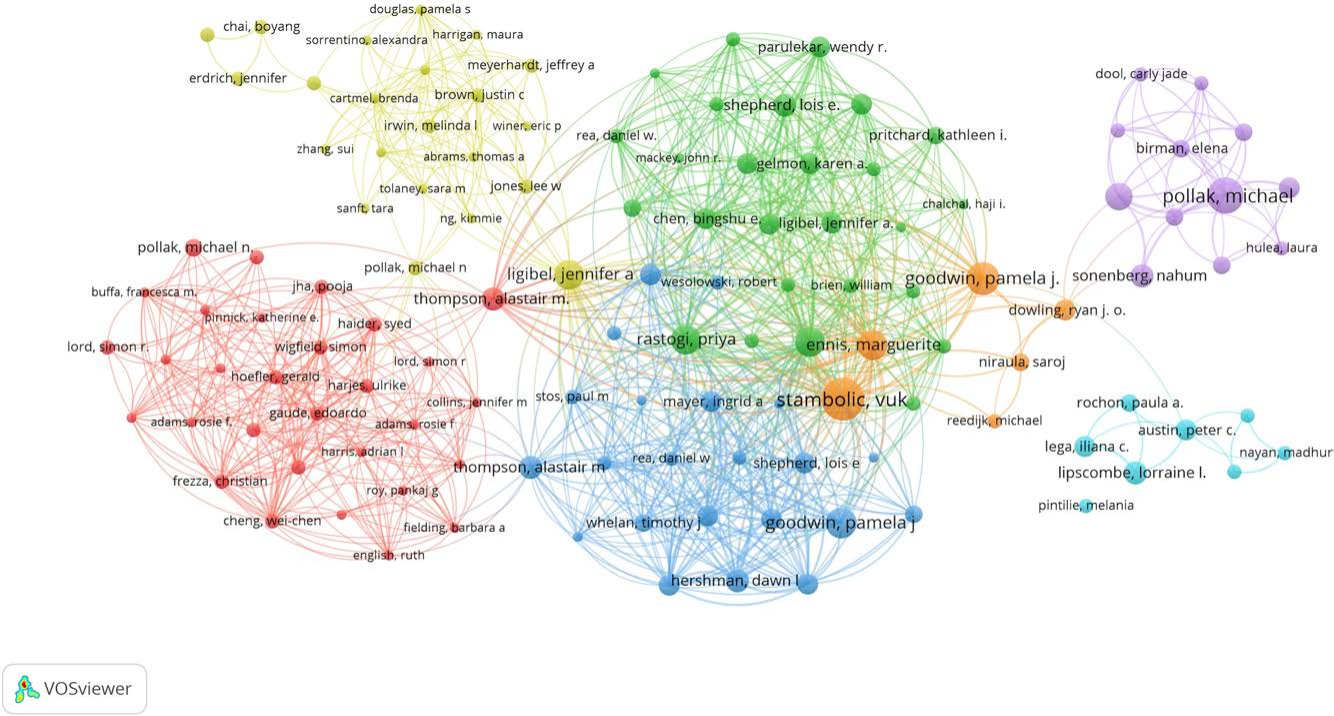
Collaborative connections among authors.

Figure 7 shows the researchers with higher central degrees in research, and Table 2 provides details on the top 10 journals in terms of publication rates. Of the 1080 entries, Table 3 lists the top 10 most cited papers worldwide. As can be seen, most of the background information regarding the relationship between diabetes and breast cancer comes from high-value authoritative journals. Among these, Pernicova I (2014) and Nat Rev Endocrinol completely wiped out the dust, with an average annual total of 86.27 citations. The top 5 journals in the core area of publications in this field were Breast Cancer Research and Treatment (3.9), PLOS ONE (3.3), Oncotarget (5.3), Cancers (4.9), and BMC Cancer (3.4). None of these journals had an IF factor of >10, indicating that the field has not received much attention and still has many questions to explore.

**Table 2:** Top 10 journals with the highest publication numbers.

	Sources	Articles
1	Breast Cancer Research and Treatment	34
2	PLOS ONE	26
3	Oncotarget	24
4	Cancers	19
5	BMC Cancer	16
6	Cancer Research	16
7	Diabetes Care	16
8	Oncology Letters	15
9	Journal of Clinical Oncology	14
10	Journal of Clinical Oncology: Official Journal of the American Society of Clinical Oncology	13

**Table 3:** Top 10 most cited references.

	Paper	Doi	Total citations	TC per yr
1	Pernicova I, 2014, Nat Rev Endocrinol	10.1038/nrendo.2013.256	949	86.27
2	Hirsch HA, 2009, Cancer Res	10.1158/0008-5472.CAN-09-2994	906	56.63
3	Currie CJ, 2009, Diabetologia	10.1007/s00125-009-1440-6	887	55.44
4	Pollak M, 2012, Nat Rev Cancer	10.1038/nrc3215	885	68.08
5	Zakikhani M, 2006, Cancer Res	10.1158/0008-5472.CAN-06-1500	883	46.47
6	Vigneri P, 2009, Endocr-relat Cancer	10.1677/ERC-09-0087	803	50.19
7	Dowling RJO, 2007, Cancer Res	10.1158/0008-5472.CAN-07-2310	770	42.78
8	Bowker SL, 2006, Diabetes Care	10.2337/diacare.29.02.06.dc05-1558	767	40.37
9	Jiralerspong S, 2009, J Clin Oncol	10.1200/JCO.2009.19.6410	708	44.25
10	Noto H, 2012, PLOS ONE	10.1371/journal.pone.0033411	455	35

TC = total compensation.

**Figure 8.**
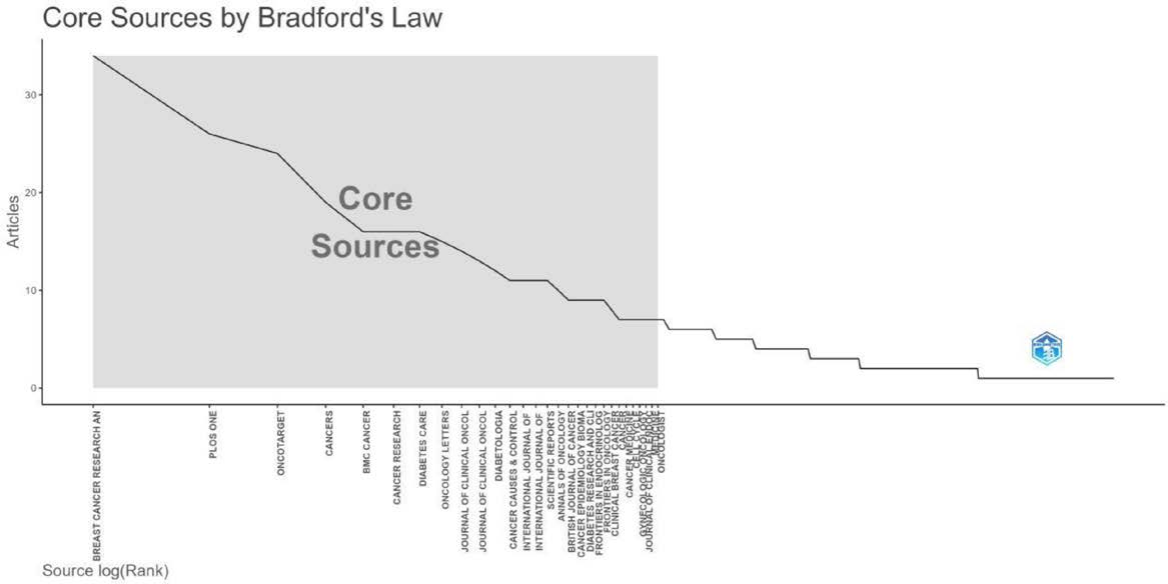
Core sources by Bradford law.

Core journals related to online literature in this field were determined based on Bradford law (Figure 8). Table 4 lists the top 10 most frequently cited, co-cited journals. Diabetes Care was the most cited, mentioned 517 times, followed by Cancer Research and Journal of Clinical Oncology. The top 3 co-cited journals in terms of centrality are the British Journal of Cancer (0.04), Breast Cancer Research and Treatment (0.02), and PLOS ONE (0.02). Co-cited references refer to the references that are cited by 2 or more publications at the same time. We carefully analyzed the top 10 citations according to the expected citation rate, as shown in Table S2, Supplemental Digital Content, https://links.lww.com/MD/R472. The study conducted by Jiralerspong *S* was cited the most, obtaining 105 citations.

**Table 4:** Top 10 most co-cited journals.

Rank	Co-cited journal	Yr	Count	Centrality	IF (2023)	JCR
1	Diabetes Care	2005	517	0	14.8	Q1
2	Cancer Research	2004	487	0	12.5	Q1
3	Journal of Clinical Oncology	2004	451	0.01	42.1	Q1
4	Breast Cancer Research and Treatment	2006	377	0.02	3	Q3
5	Diabetologia	2009	359	0	8.4	Q1
6	PLOS ONE	2010	347	0.02	2.9	Q3
7	International Journal of Cancer	2004	333	0.01	5.7	Q2
8	BMJ-British Medical Journal	2006	318	0.02	93.7	Q1
9	Cell Cycle	2009	312	0.01	3.4	Q3
10	British Journal of Cancer	2004	305	0.04	6.4	Q1

IF = impact factor, JCR = journal citation reports.

### 3.6. Analysis of frontiers and hotspots

Keyword clustering refers to the process of categorizing keywords of similar topics into multiple groups, whereas co-occurrence analysis helps to identify the relationships between various topics within a discipline. In this study, keyword co-occurrence and clustering analysis were performed to identify research hotspots and thematic structures in the field of antidiabetic drugs and breast cancer. Keywords were clustered using the log-likelihood ratio algorithm, resulting in a network composed of 582 nodes (keywords) and 3814 edges (co-occurrence links), with a network density of 0.0226. The largest connected component contained 576 nodes, accounting for 98% of the network, indicating a well-connected knowledge structure.

The clustering quality was evaluated using modularity (*Q*) and weighted mean silhouette (*S*) values. The modularity *Q* value was 0.4206, which exceeds the threshold of 0.3, suggesting a significant cluster structure. The weighted mean silhouette *S* value was 0.7328, reflecting good internal consistency within clusters. The harmonic mean of *Q* and *S* was 0.5344, further confirming the robustness and reliability of the clustering results.

These metrics indicate that the keyword clusters are clearly defined and thematically coherent, thereby facilitating a meaningful interpretation of research trends. As shown in the keyword evolution graphs (Figures S1–S4, Supplemental Digital Content, https://links.lww.com/MD/R472), the field initially focused on themes, such as insulin resistance, prevention, and endometrial cancer, with gradual shifts toward mechanisms involving estrogen receptors, phosphatidylinositol 3-kinase, and glucose metabolism. High-frequency keywords such as “risk” (222 occurrences) and “growth” (126 occurrences) underscore the research emphasis on the risks of diabetic medications and their effects on tumor growth (Table S3, Supplemental Digital Content, https://links.lww.com/MD/R472). Together, the clustering parameters and keyword trends illustrate the evolving research landscape from association studies to mechanistic investigations of metformin in breast cancer.

## 4. Discussion and conclusion

Through bibliometric analysis and in combination with a review of relevant literature, we found that metformin is the most prominent diabetic drug for the prevention or treatment of breast cancer in various studies. Therefore, exploring the most appropriate dose of metformin for inhibiting breast cancer cells has become the focus of researchers in this field.

Experimental studies support its anticancer potential,^[14,15]^ involving mechanisms such as AMPK activation and insulin level modulation.^[16–18]^ Animal models have also confirmed its ability to suppress tumor development.^[19]^ Notably, some observational studies suggest an association between cumulative metformin dose and reduced risk of specific breast cancer subtypes (e.g., HR^+^/HER2^−^), with higher-dose groups (e.g., >500 mg/day) showing a stronger potential protective trend.^[20]^

However, the evidence in this field remains inconsistent.^[21–24]^ A critical issue is that several in vitro studies use drug concentrations far exceeding the achievable plasma levels in patients with diabetes (sometimes by 10–100-fold), limiting direct clinical translation.^[25–28]^ A recent large-scale Phase III randomized trial also did not demonstrate significant survival benefits when metformin was used as an adjuvant therapy in breast cancer.^[29]^ Furthermore, drug resistance poses a challenge, with studies indicating that sustained activation of pathways such as Akt/Snail1/E-cadherin may be involved.^[30–32]^

Beyond metformin, other antidiabetic agents have also demonstrated research potential. For instance, the Sodium-glucose cotransporter 2 inhibitor ipragliflozin can inhibit breast cancer cell proliferation at concentrations of 1 to 10 μM, which is close to its pharmacological blood concentration.^[33]^ The Dipeptidyl peptidase-4 inhibitor sitagliptin has also been associated with potential breast cancer prevention after long-term use. These findings provide clues for expanding therapeutic options.^[34]^

This study employed bibliometric methods to systematically analyze research trends in the relationship between breast cancer and antidiabetic drug dosage, mapping the evolution of hotspots and knowledge structure, to provide insights for future research directions. This study has some limitations: this interdisciplinary field is still developing, and the volume of available literature is limited; reliance on the WOSCC and PubMed databases may affect the coverage of conclusions; and the bibliometric software tools used have inherent methodological constraints. Future well-designed prospective clinical studies, particularly those focusing on different dosing regimens and long-term effects in specific populations, are needed to further validate and deepen the current understanding.

## Acknowledgments

We thank Editage (www.editage.cn) for English language editing.

## Author contributions

**Conceptualization:** Zhaoning Wang, Fangrui Zhao, Linlin Wang.

**Data curation:** Zhaoning Wang.

**Formal analysis:** Zhaoning Wang.

**Methodology:** Zhaoning Wang, Fangrui Zhao, Linlin Wang.

**Funding acquisition:** Linlin Wang.

**Writing – original draft:** Zhaoning Wang.

**Writing – review & editing:** Fangrui Zhao, Linlin Wang.

## Supplementary Material


