# Supplementary material for: Relationship between breast cancer and metformin: A bibliometric review
Source: Medicine (Baltimore). 2026 Feb 28;105(9):e47868. doi: 10.1097/MD.0000000000047868 (PMC12956224; doi:10.1097/MD.0000000000047868)
Supplement: Supplementary file 1 [file medi-105-e47868-s001.docx]

Supplementary Table.1 Literature search criteria

PubMed

**(("Metformin"[Mesh] OR "Metformin"[Title/Abstract] OR "Dimethylbiguanide"[Title/Abstract] OR "Dimethylguanylguanidine"[Title/Abstract] OR "Glucophage"[Title/Abstract] OR "Metformin Hydrochloride"[Title/Abstract] OR "Hydrochloride, Metformin"[Title/Abstract] OR "Metformin HCl"[Title/Abstract] OR "HCl, Met"[Title/Abstract]) AND ("Diabetes Mellitus"[Mesh] OR "Diabetes"[Title/Abstract] OR "Diabetes Mellitus"[Title/Abstract] OR "Hyperglycemia"[Title/Abstract] OR "Hyperglycaemia"[Title/Abstract])) AND ("Breast Neoplasms"[Mesh] OR "Breast Neoplasm"[Title/Abstract] OR "Neoplasm, Breast"[Title/Abstract] OR "Neoplasms, Breast"[Title/Abstract] OR "Breast Tumors"[Title/Abstract] OR "Breast Tumor"[Title/Abstract] OR "Tumor, Breast"[Title/Abstract] OR "Tumors, Breast"[Title/Abstract] OR "Breast Cancer"[Title/Abstract] OR "Cancer, Breast"[Title/Abstract] OR "Cancer of Breast"[Title/Abstract] OR "Cancer of the Breast"[Title/Abstract] OR "Malignant Neoplasm of Breast"[Title/Abstract] OR "Breast Malignant Neoplasm"[Title/Abstract] OR "Breast Malignant Neoplasms"[Title/Abstract] OR "Malignant Tumor of Breast"[Title/Abstract] OR "Breast Malignant Tumor"[Title/Abstract] OR "Breast Malignant Tumors"[Title/Abstract] OR "Mammary Cancer"[Title/Abstract] OR "Cancer, Mammary"[Title/Abstract] OR "Cancers, Mammary"[Title/Abstract] OR "Mammary Cancers"[Title/Abstract] OR "Mammary Neoplasms, Human"[Title/Abstract] OR "Human Mammary Neoplasm"[Title/Abstract] OR "Human Mammary Neoplasms"[Title/Abstract] OR "Neoplasm, Human Mammary"[Title/Abstract] OR "Neoplasms, Human Mammary"[Title/Abstract] OR "Mammary Neoplasm, Human"[Title/Abstract] OR "Breast Carcinoma"[Title/Abstract] OR "Breast Carcinomas"[Title/Abstract] OR "Carcinoma, Breast"[Title/Abstract] OR "Carcinomas, Breast"[Title/Abstract] OR "Mammary Carcinoma, Human"[Title/Abstract] OR "Carcinoma, Human Mammary"[Title/Abstract] OR "Carcinomas, Human Mammary"[Title/Abstract] OR "Human Mammary Carcinomas"[Title/Abstract] OR "Mammary Carcinomas, Human"[Title/Abstract] OR "Human Mammary Carcinoma"[Title/Abstract])**

**Web of Science**

**TS=("Metformin" OR "Dimethylbiguanide" OR "Dimethylguanylguanidine" OR "Glucophage" OR "Metformin Hydrochloride" OR "Hydrochloride, Metformin" OR "Metformin HCl" OR "HCl, Met") AND TS=("Diabetes Mellitus" OR "Diabetes" OR "Hyperglycemia" OR "Hyperglycaemia") AND TS=("Breast Neoplasms" OR "Breast Neoplasm" OR "Neoplasm, Breast" OR "Neoplasms, Breast" OR "Breast Tumors" OR "Breast Tumor" OR "Tumor, Breast" OR "Tumors, Breast" OR "Breast Cancer" OR "Cancer, Breast" OR "Cancer of Breast" OR "Cancer of the Breast" OR "Malignant Neoplasm of Breast" OR "Breast Malignant Neoplasm" OR "Breast Malignant Neoplasms" OR "Malignant Tumor of Breast" OR "Breast Malignant Tumor" OR "Breast Malignant Tumors" OR "Mammary Cancer" OR "Cancer, Mammary" OR "Cancers, Mammary" OR "Mammary Cancers" OR "Mammary Neoplasms, Human" OR "Human Mammary Neoplasm" OR "Human Mammary Neoplasms" OR "Neoplasm, Human Mammary" OR "Neoplasms, Human Mammary" OR "Mammary Neoplasm, Human" OR "Breast Carcinoma" OR "Breast Carcinomas" OR "Carcinoma, Breast" OR "Carcinomas, Breast" OR "Mammary Carcinoma, Human" OR "Carcinoma, Human Mammary" OR "Carcinomas, Human Mammary" OR "Human Mammary Carcinomas" OR "Mammary Carcinomas, Human" OR "Human Mammary Carcinoma")**

Supplementary Table.2 Top ten most co-cited reference

| Rank | Authors | References | Citation | Centrality |
| --- | --- | --- | --- | --- |
| 1 | Jiralerspong S | Metformin and pathologic complete responses to neoadjuvant chemotherapy in diabetic patients with breast cancer | 105 | 0.01 |
| 2 | Currie CJ | The influence of glucose-lowering therapies on cancer risk in type 2 diabetes | 84 | 0.03 |
| 3 | Libby G | New users of metformin are at low risk of incident cancer: a cohort study among people with type 2 diabetes | 80 | 0.03 |
| 4 | Bodmer M | Long-term metformin use is associated with decreased risk of breast cancer | 67 | 0.02 |
| 5 | DeCensi A | Metformin and cancer risk in diabetic patients: a systematic review and meta-analysis | 66 | 0.13 |
| 6 | Landman GWD | Metformin associated with lower cancer mortality in type 2 diabetes: ZODIAC-16 | 63 | 0.09 |
| 7 | Hirsch HA | Metformin selectively targets cancer stem cells, and acts together with chemotherapy to block tumor growth and prolong remission | 62 | 0.02 |
| 8 | Alimova IN | Metformin inhibits breast cancer cell growth, colony formation and induces cell cycle arrest in vitro | 61 | 0.02 |
| 9 | Currie CJ | Mortality after incident cancer in people with and without type 2 diabetes: impact of metformin on survival | 59 | 0.01 |
| 10 | Zakikhani M | Metformin is an AMP kinase–dependent growth inhibitor for breast cancer cells | 53 | 0 |

Supplementary Table.3 The twenty keywords with highest frequency

| Rank | Keywords | Centrality | Year | Count |
| --- | --- | --- | --- | --- |
| 1 | breast cancer | 0.04 | 2004 | 560 |
| 2 | risk | 0.05 | 2006 | 222 |
| 3 | metformin | 0.04 | 2004 | 165 |
| 4 | diabetes mellitus | 0.15 | 2007 | 148 |
| 5 | growth | 0.08 | 2004 | 126 |
| 6 | activated protein kinase | 0.13 | 2006 | 106 |
| 7 | insulin | 0.05 | 2009 | 99 |
| 8 | mortality | 0.05 | 2008 | 99 |
| 9 | prostate cancer | 0.06 | 2010 | 97 |
| 10 | pancreatic cancer | 0.08 | 2006 | 93 |
| 11 | mellitus | 0.05 | 2006 | 88 |
| 12 | colorectal cancer | 0.06 | 2006 | 83 |
| 13 | survival | 0.05 | 2011 | 73 |
| 14 | metaanalysis | 0.04 | 2009 | 70 |
| 15 | in vitro | 0.07 | 2009 | 70 |
| 16 | type 2 diabetes mellitus | 0.08 | 2009 | 64 |
| 17 | expression | 0.08 | 2008 | 61 |
| 18 | association | 0.08 | 2009 | 57 |
| 19 | insulin resistance | 0.1 | 2004 | 55 |
| 20 | therapy | 0.06 | 2006 | 52 |

**Supplemental Figures:**


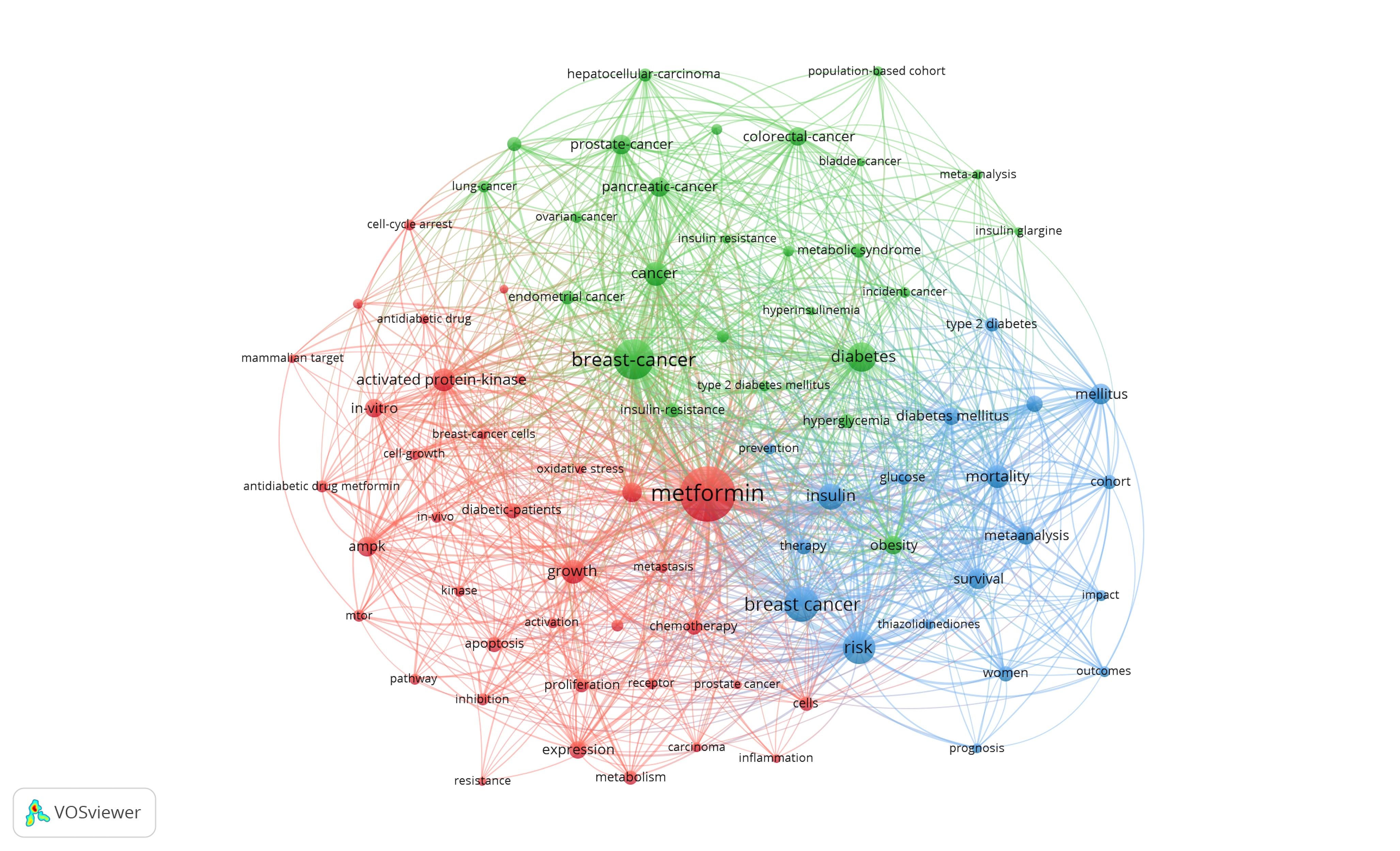


Supplementary Fig.1 Cluster of keywords


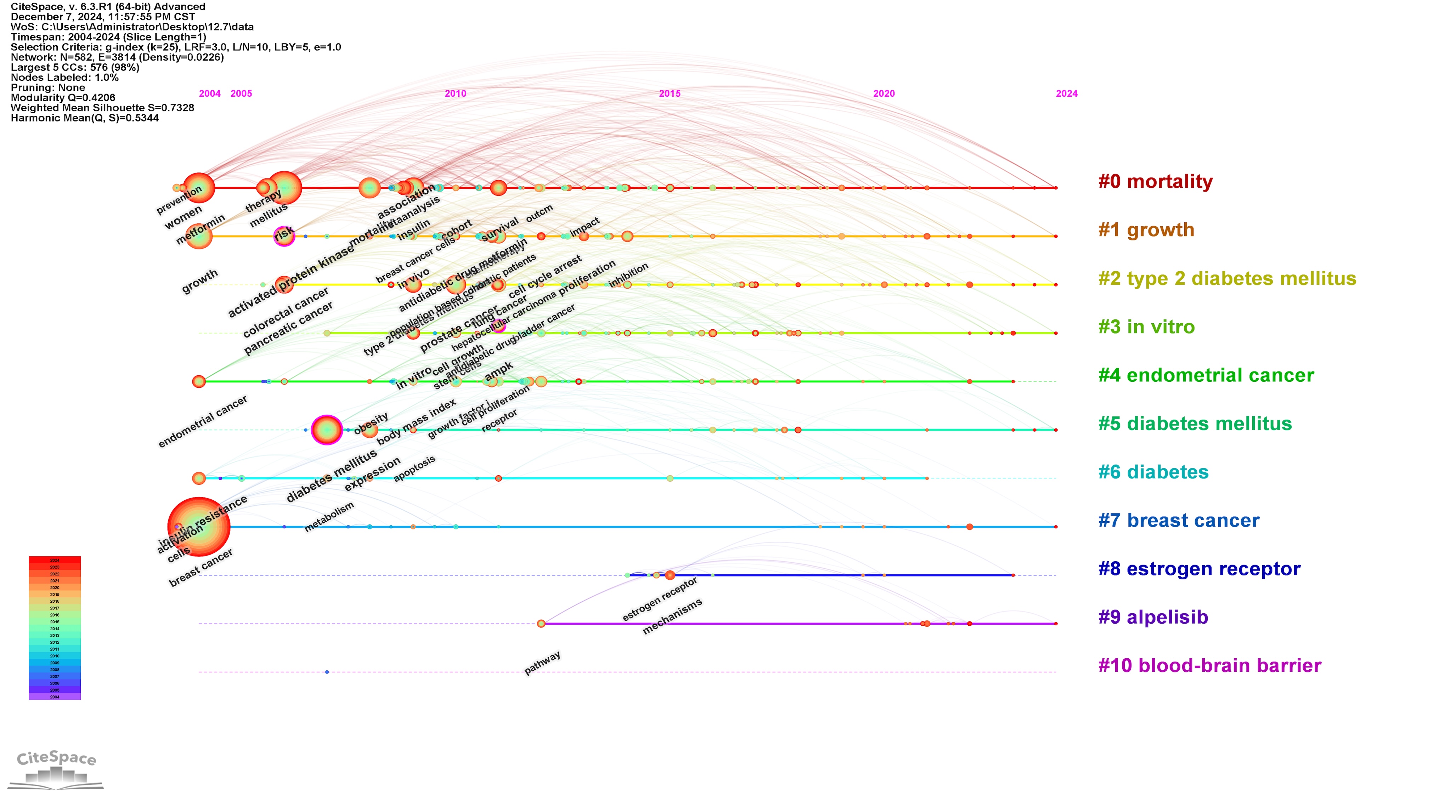


Supplementary Fig.2 Keyword timezone map


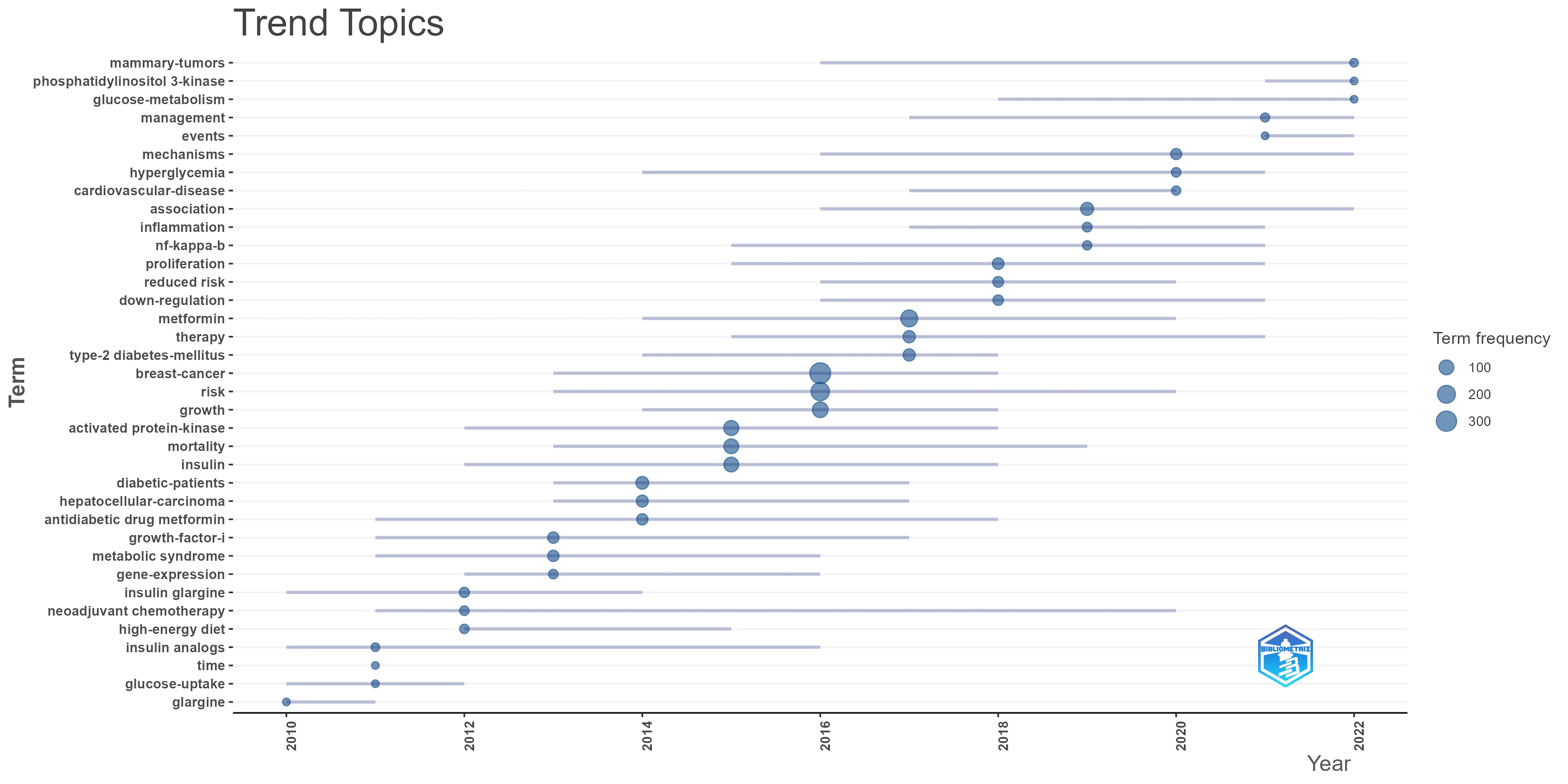


Supplementary Fig.3 Trend topics


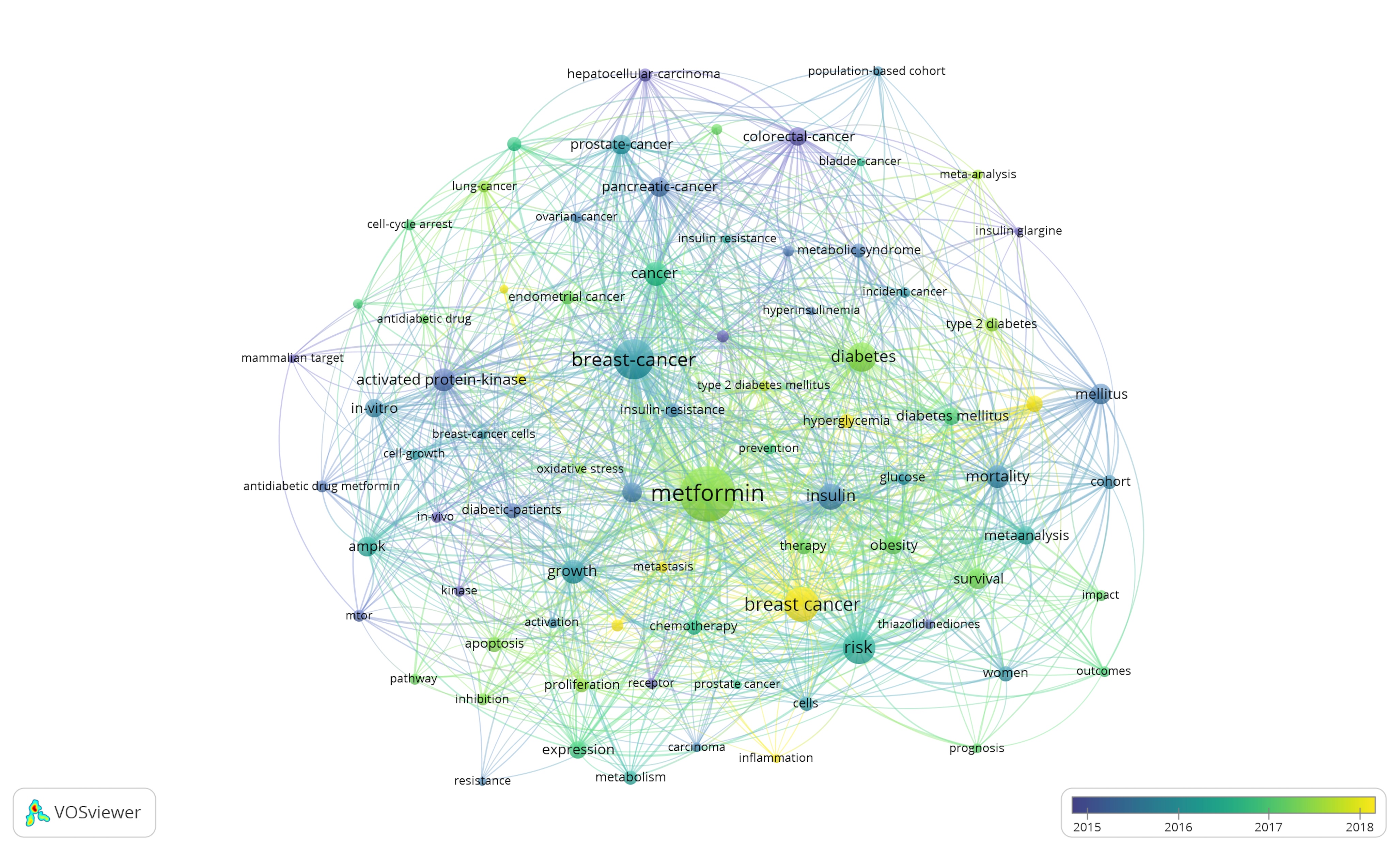


Supplementary Fig.4 Emporal changes in keyword clustering
